# Inference of molecular orientation/ordering change nearby topological defects by the neural network function from the microscopic color information

**DOI:** 10.1038/s41598-021-88535-7

**Published:** 2021-04-27

**Authors:** Haruka Sakanoue, Yuki Hayashi, Kenji Katayama

**Affiliations:** Department of Applied Chemistry, Chuo University, Tokyo, 112-8551 Japan

**Keywords:** Physical chemistry, Imaging techniques, Microscopy, Topological defects, Chemical physics

## Abstract

Topological defects in liquid crystals (LCs) dominate molecular alignment/motion in many cases. Here, the neural network (NN) function has been introduced to predict the LC orientation condition (orientation angle and order parameter) at local positions around topological defects from the phase/polarization microscopic color images. The NN function was trained in advance by using the color information of an LC in a planar alignment cell for different orientation angles and temperatures. The photo-induced changes of LC molecules around topological defects observed by the time-resolved measurement was converted into the image sequences of the orientation angle and the order parameter change. We found that each pair of brushes with different colors around topological defects showed different orientation angle and ordering changes. The photo-induced change was triggered by the photoisomerization reaction of molecules, and one pair of brushes increased in its order parameter just after light irradiation, causing gradual rotation in the brush. The molecules in the other pair of brushes were disordered and rotated by the effect of the initially affected region. This combination approach of the time-resolved phase/polarization microscopy and the NN function can provide detailed information on the molecular alignment dynamics around the topological defects.

## Introduction

Topological defects in liquid crystals (LCs) have been paid much attention both in basic science and technological applications. They were also used for the analogical system for the cosmological theory^1,2^. They are the structural singular points of the LC alignment, and there are a variety of defect types, which are categorized according to the topological charge. These topological defects can be spontaneously formed when LCs are put into a glass cell without an alignment layer. Due to the high elastic energy at these defect positions, they are thermodynamically intermediate states and will disappear for some duration of time.

These days, the molecular alignment of LCs can be controlled by various techniques, and topological defects can be intentionally generated by the patterning of an alignment layer^3–5^. Many types of thin optics with topological defects using the LC patterns were demonstrated using a photo-alignment layer^6–8^. Topological defects also could be formed by stabilizing the alignment by a polymer pattern without a photo-alignment layer^9^. Many interesting phenomena relevant to the topological defects were reported in biology, too. The self-propelled bacteria's motion was controlled by the LC patterns, including topological defects formed on a photo-alignment layer^10^. It was reported that the cell growth and the collective motion were controlled by the topological defects formed by the biological cells aligned like LC molecules, and the fate of the cells was determined by the type of the topological defects^11,12^, and predesigned cell culture on a photoaligned LC elastomer was demonstrated^13^. Those studies indicate that the collective motion of objects and molecules is dominated by topological defects, and the underlying principle should be studied.

On the other hand, we have investigated the photoinduced behavior of LCs, including photo-responsive molecules. We prepared an LC-made double emulsion, and the photo-induced phase transition was observed, and the molecular orientation changed from the center of a topological defect^14^. Also, we demonstrated the photo-induced rolling motion of an LC droplet in a surfactant solution and found that the topological defect was oriented toward the light source^15^. In the previous paper, we studied the molecular orientation change around topological defects by using the polarization/phase microscopy and found that the molecular orientation and the ordering changes occurred step-by-step instead of changing them at the same time^16^. These studies indicate that the molecular orientation change around topological defects seems more complicated than believed.

This study introduced the neural network (NN) prediction of the orientation conditions of LC (orientation angle and order parameter) with a microscopic resolution. Microscopic prediction of the orientation conditions was studied under the ultrasound excitations, and the orientation was calculated from the local transmittance of the microscopic image, providing a three-dimensional molecular orientation^17^. Also, there were several applications using NN for LCs; it was used for temperature estimation by using the color information of LCs^18^; convolutional NN for image analysis^19^ was used for detection of topological defects, and the topological dynamics were studied^20^; several physical properties such as the order parameter, temperature and pitch length of cholesteric LCs were predicted from the patterns of LCs^21,22^. In this study, by training the NN function by using the color information for different orientation angles and temperatures in a planer cell in advance, we could predict the molecular orientation angle and the order parameter from the local color information. By inducing a perturbation on the LCs by photo-irradiation, the change of the molecular orientation/ordering change around the topological defects was observed. We could clarify the complicated processes of the molecular alignment change from the time sequence of the molecular orientation angle and the order parameter.

## Microscopic observation and neural network prediction

The color difference of LCs observed by a microscope is used to obtain different orientation angles and ordering conditions by the distinction of the LC conditions using a pre-trained NN function. We used a microscope with a combination of the polarized and phase-contrast functions to have a color difference for different orientation angles and ordering conditions. The polarization microscope is usually used to observe the LC orientation angle, where the LC orientation angle at 45 degrees to the polarizer and analyzer shows the highest transmission. However, the polarized microscope cannot distinguish the molecular orientation angles parallel or perpendicular to the polarizer/analyzer. When we use the phase-contrast microscope to observe LCs, we could distinguish a different angle and ordering conditions from different color regions because the extraordinary and ordinary refractive indexes contribute, but the information on the orientation direction is unclear. The color depends both on the orientation direction and the ordering of LCs by a combination of polarization and phase-contrast microscopy. The ratio of the transmittance for the extraordinary and ordinary directions is varied by a change in the orientation angle. As a result, the final color depends on the angle. Furthermore, the molecular ordering change causes the refractive index change both in the extraordinary and ordinary directions, and the final color also depends on it.

Although the final transmittance can be theoretically predicted based on optics theory^16^, (Appendix in Supporting Information (SI)) the absolute transmittance is necessary, and it needs a careful procedure to calibrate the light intensities of transmittance. This procedure was troublesome for LCs with a transmittance spectrum, including oscillation due to the light interference for a thin LC layer, which is easily affected by the variation of LC cells. However, we could obtain only three observables, RGB values detected with a color camera. This is why the NN function was introduced for the empirical estimation of the orientation angle and ordering.

An optical configuration of the device is the same as before^16^ (Figure S1 and its description in Supporting Information (SI)). Briefly, the microscope consisted of a phase microscope with a polarization-dependent detection, and the spatial resolution was around 1 µm. A sample was illuminated by a UV-LED, and the photo-induced change was observed by a CMOS camera using a white light illumination. A sample is subject to photo-isomerization by the UV light, causing the molecular orientation change. The UV light was illuminated to the sample for 200 ms and turned off. The image sequence was obtained at an interval of 20 or 30 ms.

**Figure 1:**
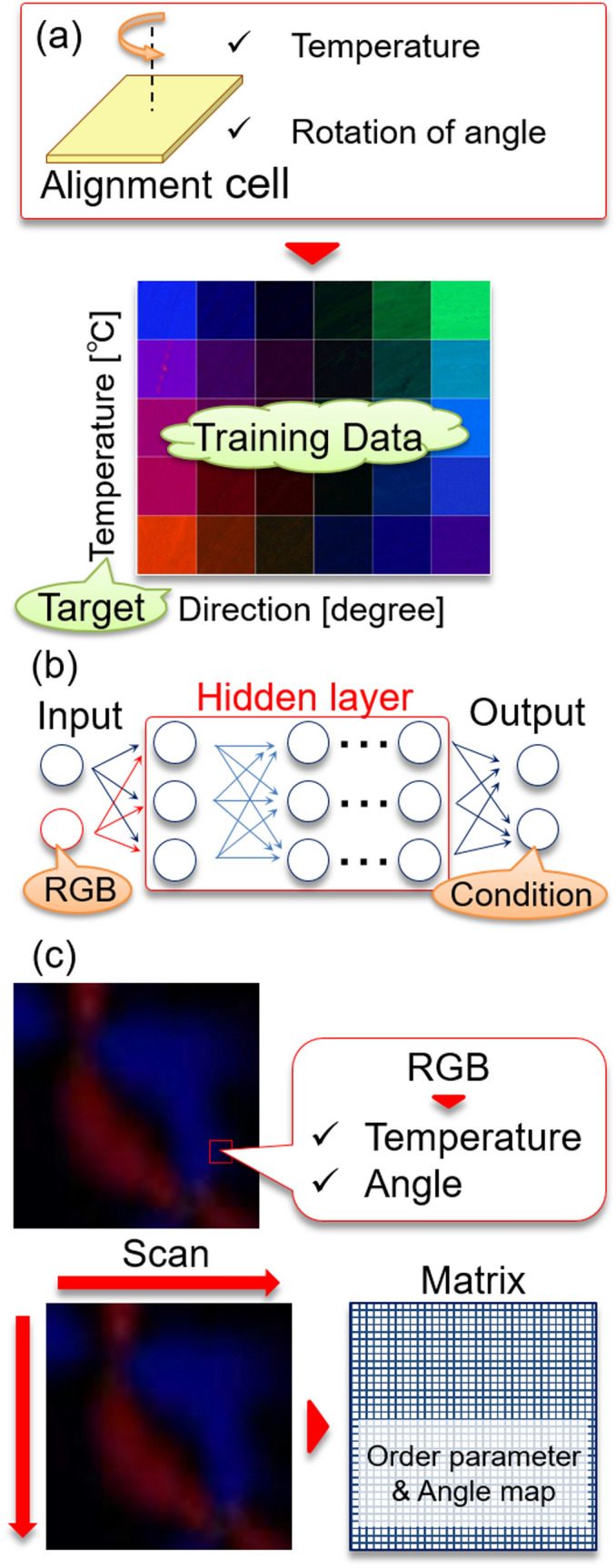
The summary of the methodology of predicting the orientation angle and the temperature by using the neural network function is shown. **(a)** The color information for different angles and temperatures in a planer alignment cell was collected. **(b)** The neural network function was trained by the RGB data to provide the orientation angle and temperature. **(c)** At each local position, the color information is converted into the temperature and angle. This calculation was performed for all the pixels of images by scanning, and the data was changed into the order parameter and angle map (prepared by PowerPoint 2016).

**Figure 2:**
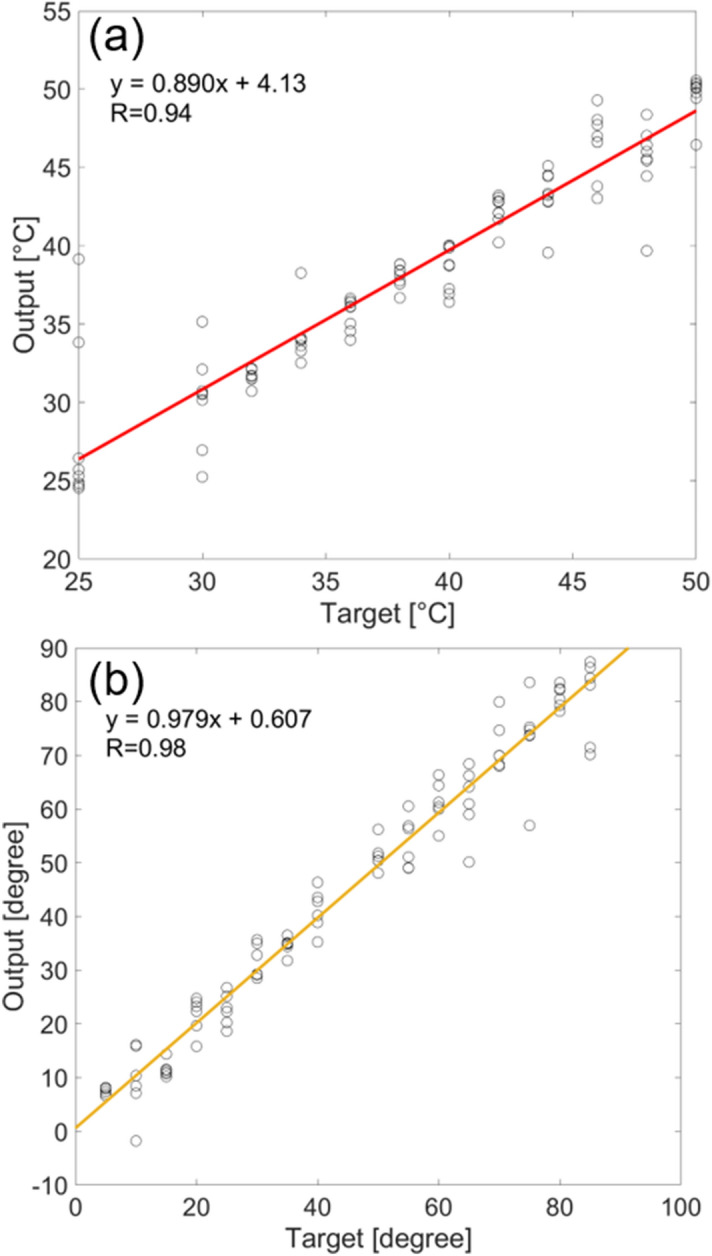
The neural network response fittings for the training data of the temperature **(a)** and the orientation angle, **(b)**. The target values correspond to the experimental conditions of the angles and the temperatures. The correlation coefficients, R, are shown for reference.

Next, the NN prediction procedure for the orientation angle and the order parameter is described in Fig. 1. An LC in an alignment cell was measured under various temperatures and angle conditions and obtain the color information for each condition. This information was used for the training of a NN function. The alignment cell was rotated at every 5 degrees, and the temperature was changed from 25 to 50 degrees at an interval of 2 or 5 degrees, and the color was measured for each condition. The NN function was trained to predict the orientation angle and the temperature from the color information (RGB values), with the layer number of 20 and the optimal epoch of 12. We used 96 combinations of the temperatures and angles dataset. The correlation plot for the temperature and the orientation angle is shown in Fig. 2. The correlation coefficients were sufficiently high to predict the orientation angle and temperature as long as the angles and temperatures range in the training data. At each local position of a microscopic image, the RGB information could be converted into the orientation angle and the temperature, as shown in Fig. 1c.

The difference in the predicted temperatures at local positions in an image does not mean that the temperature is varied at different positions in the same sample, but indicating that the ordering condition is different. Around the topological defects, the ordering conditions were different at each local position. We assume that the local LC ordering is represented by the ordering condition at different temperatures for an LC in the alignment cell. The NN function then corresponds the color at each local position to the LC conditions in the alignment cell at a specific temperature and orientation angle. To make a correlation between the order parameter and the temperature for an LC in an alignment cell, the temperature dependence of the order parameter (*P*) was obtained using the polarized absorption measurement. The order parameter was obtained from the absorbance parallel and perpendicular to the polarizer,${A}_{\parallel }$ ${A}_{\perp }$ and was calculated as:

1 $$P=\frac{{A}_{\parallel }-{A}_{\perp }}{{A}_{\parallel }+2{A}_{\perp }}.$$

The polarized absorbance spectra were measured by changing the temperature, and an example of it is shown in Fig. S2(a) in SI. The peak wavelength of ${A}_{\parallel }$ was used for the calculation. Figure S2(b) shows the temperature dependence of P, and the fitting curve for the order parameter as a function of temperature is shown in Figure S2(b). This fitting function was used for the conversion of the temperature into the order parameter. As a final procedure, shown in Fig. 1c, the color information was converted into the temperature and angle at each local position. This calculation was performed for all the pixels of images by scanning, and the data was converted into the order parameter and angle map. This methodology allows us to measure the order parameter and the orientation angle at each local position in real-time because the microscopic color information is directly converted into the order parameter and angle map, which is different from the other techniques; the director angle is usually obtained by the polarized microscope by rotation of a sample or a polarizer.

*N*-(4-methoxybenzylidene)-4-butylaniline (MBBA, Tokyo Kasei) was used as purchased. The sample was put into an LC cell (E.H.C) with a sample thickness of 3 µm. An alignment planar cell was used to collect the training data for the NN function, and the color images were obtained at different angles of the alignment direction to the polarizer and at temperatures from 25.0 to 50.0 °C. We used an LC cell with non-rubbed polyimide layers inside for observation of topological defects, to make the same cell conditions as the training data acquisition, and the temperature was set at 30.0 °C. Under these experimental conditions, we could mostly find a pair of topological defects with + 1 and − 1 charges and found + 1/2 and − 1/2 pairs very scarcely. In this study, the pairs of ∓ 1 defects were studied. The pump light was UV-LED (SOLIS-365C, Thorlabs; wavelength: 365 nm, intensity: 0.625 mW/cm^2^), and the illumination light was white LED (SOLIS-3C, Thorlabs; intensity: 0.098 mW/cm^2^). A color CMOS camera (VCXU-23C, Baumer) was used for measurements, and a color image sequence was obtained. The temperature was controlled by a temperature controller for microscopes (TP-CHS-C, Tokai Hit). The intensity of the pump light was adjusted to prevent the phase transition.

## Result and discussions

**Figure 3:**
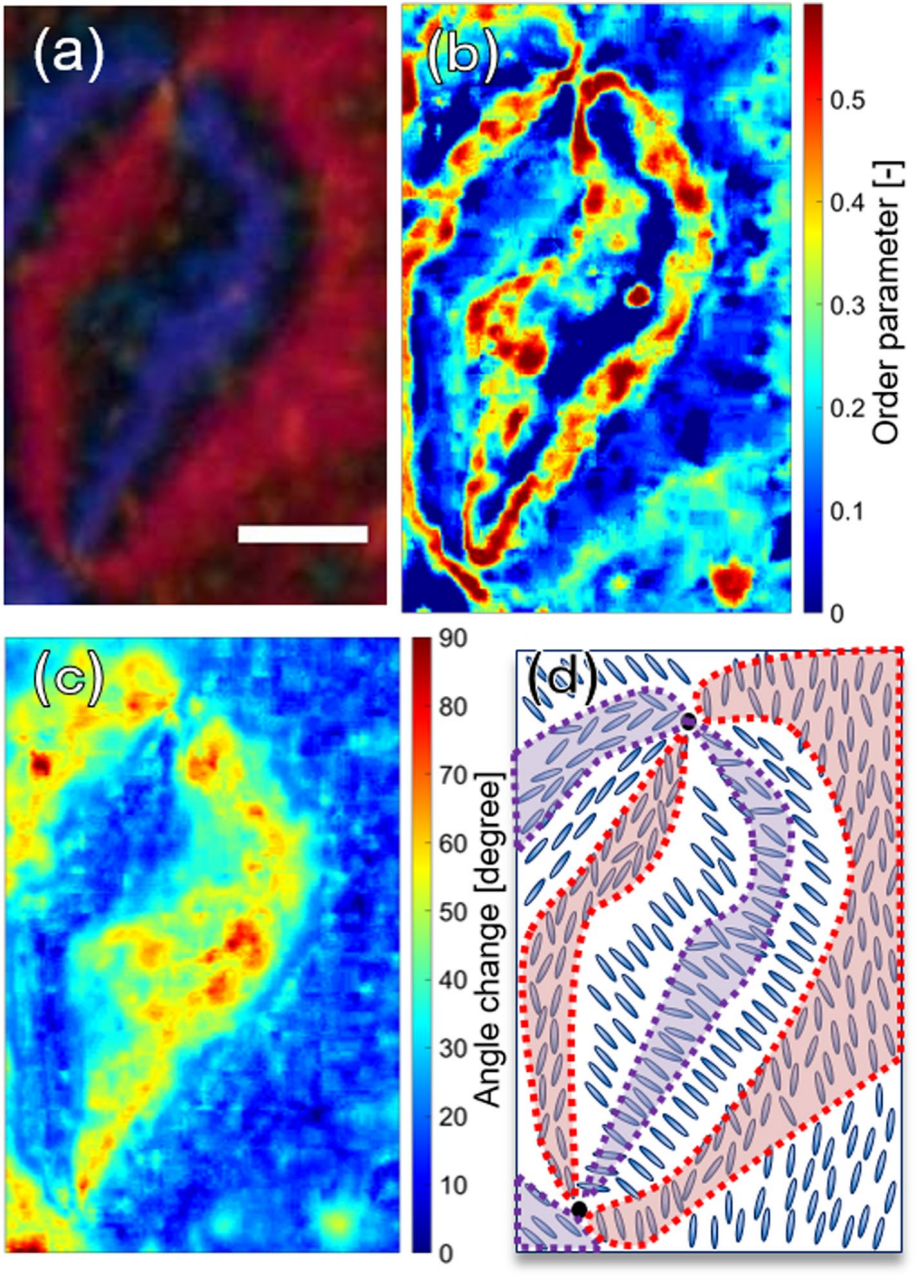
**(a)** A microscopic color image of MBBA in an LC cell before the irradiation of the UV light is shown. The scale bar corresponds to 20 μm. The predicted images of the order parameter **(b)** and the orientation angle **(c)** using the color information of **(a)** by the neural network function. **(d)** Based on **(b,c)**, the schematic drawing of the molecular ordering and orientation is shown (prepared by PowerPoint 2016).

The prediction results of the orientation angle and the order parameter using the NN function are shown in Fig. 3 for the static condition around topological defects before UV light irradiation. In the microscopic image, (a), there are two topological defects, connected by two pairs of brushes with red and purple colors. Different colors of brushes represent different order parameters and orientation angles. Both the two defects had + 1 and − 1 topological charges, confirmed from the rotation behavior by the polarizer rotation. At each local position in the image, the temperatures and the orientation angles were predicted from the color information of a. The temperature was converted to the order parameter as described in the Experimental section. The estimation maps for them are shown in b and c, respectively. In Fig. 3, the order parameter at the boundary region of the pairs of brushes (black region in a) was much higher (0.3–0.55) than those in the purple and red brushes (0–0.1). The molecular orientation angle in the purple brush was about 40 degrees different from the red brush region. In short, two colored brushes had different molecular orientation angles and less ordered than the boundary between the two pairs of brushes. A drawing of the predicted molecular alignment is summarized as shown in d.

**Figure 4:**
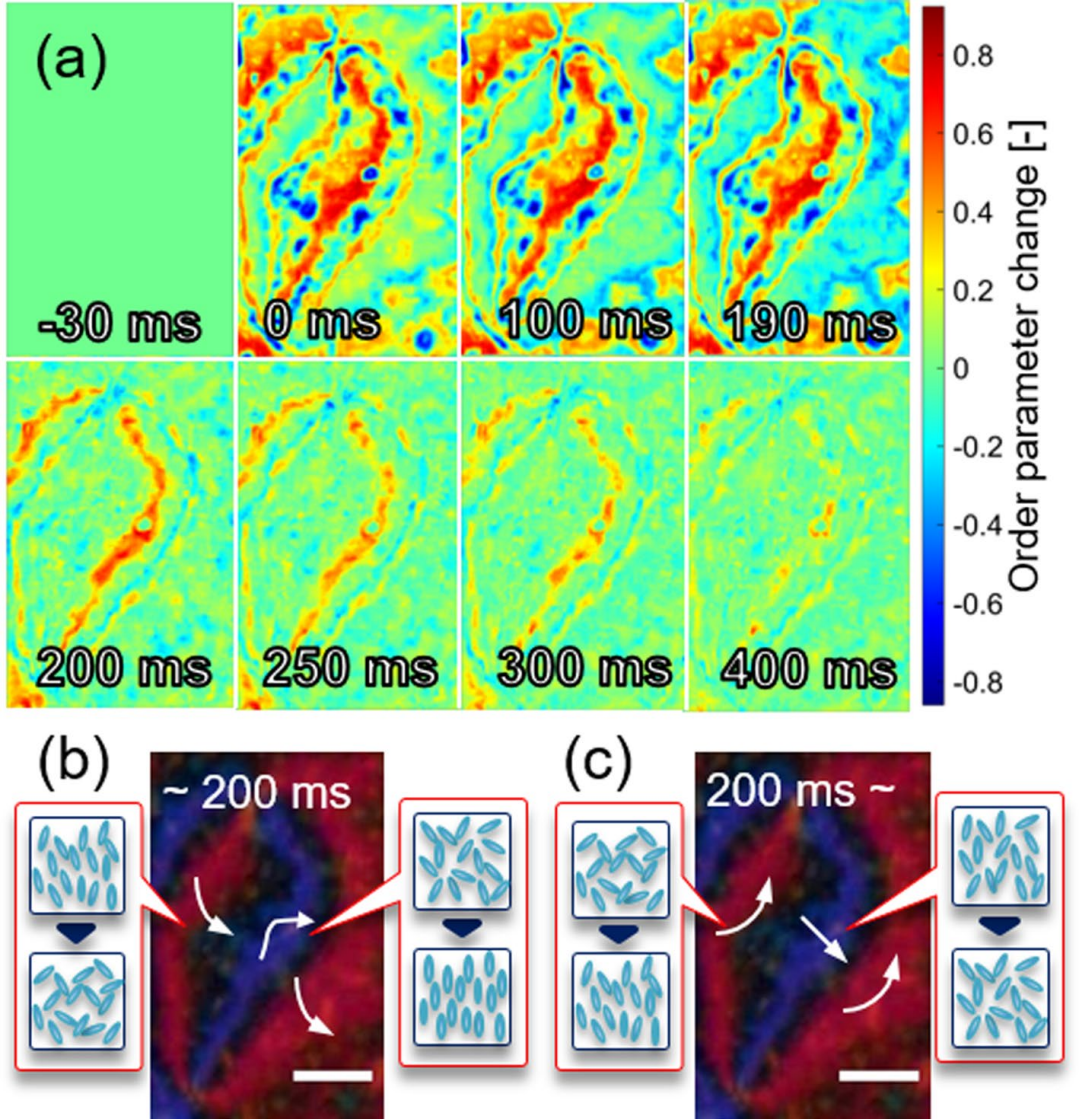
**(a)** A time sequence of the order parameter change under the UV light irradiation is shown. The UV light was irradiated from 0 to 200 ms. The difference from the original order parameter obtained in Fig. 3 is shown. **(b,c)** The summarized drawings of the change in the order parameter at each pair of brushes **(b)** during and **(c)** after light irradiation (prepared by PowerPoint 2016).

For the same region as Fig. 3, a UV light was irradiated, and the photo-induced changes of the molecular orientation and the order parameter were studied. For each frame of the movie, the order parameter and the orientation angle were predicted. The predicted image sequences of the photo-induced change were obtained by subtracting the original order parameter and orientation angle obtained in Fig. 3 from each movie frame (Subtraction image). The subtraction image sequence of the order parameter is shown in Fig. 4. The UV light was irradiated from 0 to 200 ms. The difference was negligible before the UV irradiation shown in the image 30 ms before the light irradiation. Obviously, in the purple brush in Fig. 3a, the order parameter increased instantly after light irradiation. It decreased quickly after turning off the light (200 ms), and it gradually recovered for another 200 ms. On the other hand, in the red brush in Fig. 3a, the order parameter decreased gradually during the light irradiation and recovered quickly to the original value by turning it off. In short, the light irradiation induced the ordering increase quickly in one pair of the brushes, while the other pair of brushes decreased the ordering gradually, and the light-induced effect was quickly recovered by turning off with some regions for extra time for recovery. Figure 4b,c are the summary drawings of the ordering change during and after the UV irradiation.

**Figure 5:**
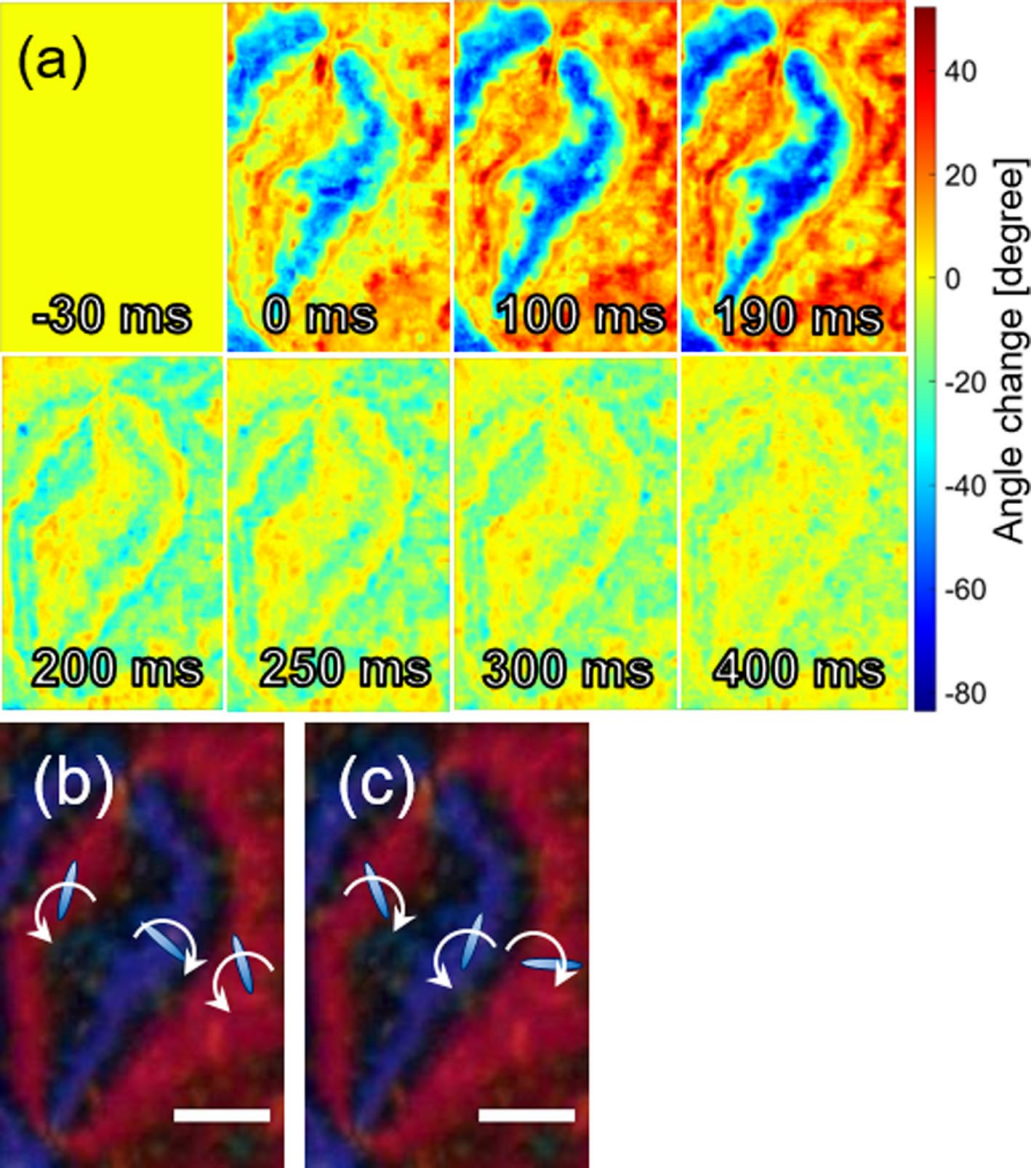
**(a)** A time sequence of the orientation angle change under the UV light irradiation. The UV light was irradiated from 0 to 200 ms. The difference from the original orientation angle obtained in Fig. 3 is shown. **(b,c)** The summarized drawings of the change in the orientation angle at each brush **(b)** during and **(c)** after light irradiation (prepared by PowerPoint 2016).

Figure 5 shows a temporal change in the orientation angle. By the UV light irradiation, the orientation angle in the purple brushes in Fig. 3a showed a clockwise rotation, while a counter-clockwise rotation was observed in the red brushes in Fig. 3a and the orientation angle change became gradually larger. This means that the opposite directional rotation happened at two domains contacting each other. The angle change recovered quickly after turning off the light, with a slight difference recovered for about another 200 ms. Figure 5b,c are the summary drawings of the molecular orientation angle change during and after the UV irradiation.

**Figure 6:**
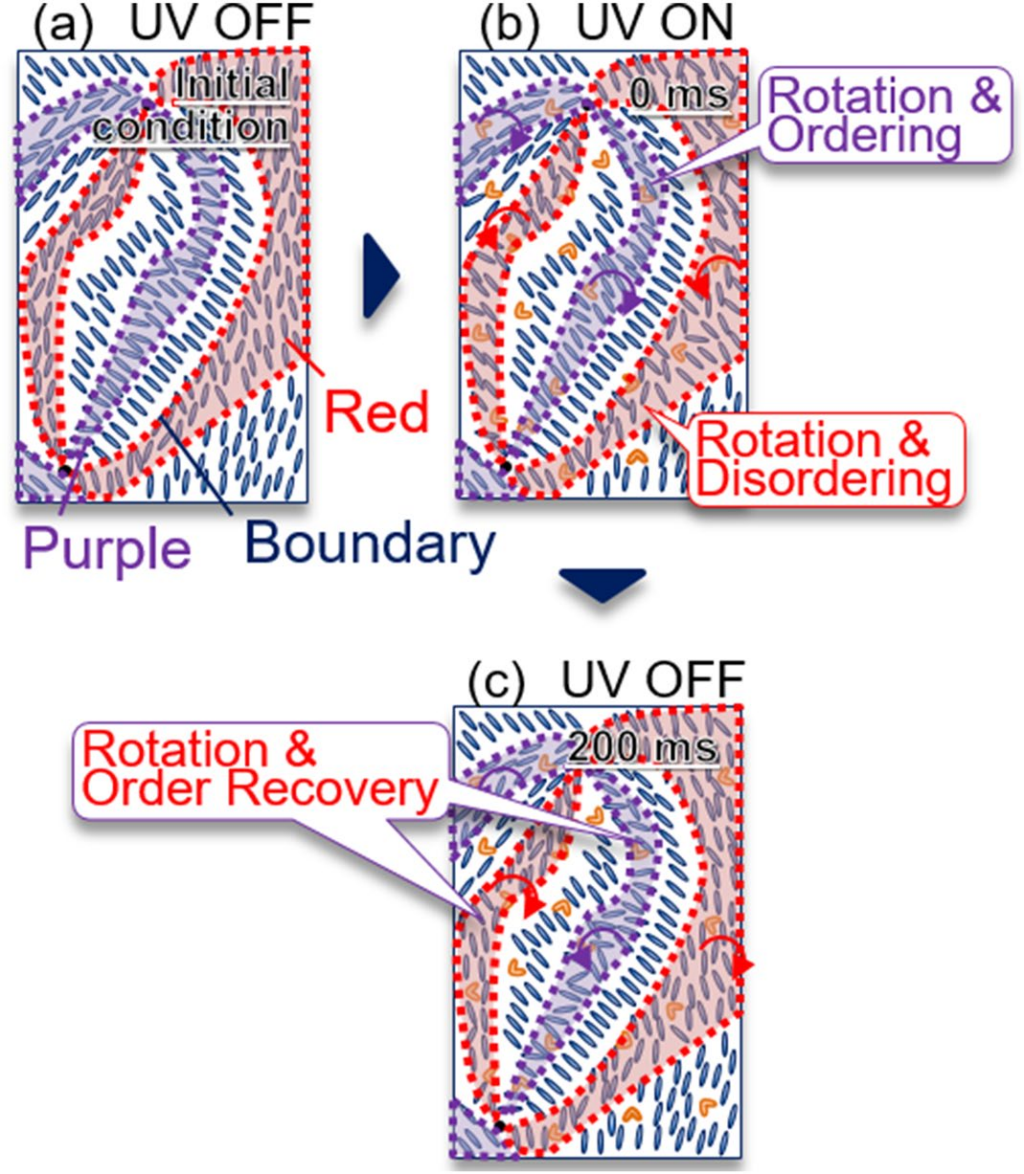
The schematic drawings of the molecular alignment change **(a)** before the light irradiation (− 20 ms), **(b) **during the irradiation (0, 190 ms), and **(c)** after irradiation (200 ms ~), The purple and red regions correspond to each colored brush (prepared by PowerPoint 2016).

Figure 6 shows a summary of the drawing of the molecular alignment change. The purple and red regions in Fig. 6a corresponded to each colored brushes in the observation image in Fig. 3a. These two colored regions correspond to the domains with different orientation angles. The photo-isomerization occurred instantly by the UV light irradiation. The molecules in the purple brushes in Fig. 3a increased the ordering and showed a counter-clockwise rotation instantly by the light irradiation, and they further gradually rotated the orientation angle, while the ordering decreased and the opposite directional rotation happened gradually in the red brush. Since the photo-induced change in the purple brush was started at first, this change seems to trigger the other changes. It is supposed that the photo-induced force due to the photo-isomerization induced a frustration force to one type of the domains, causing the ordering changed and the following rotation of the molecular orientation angle. As a result, the counterpart domain was also rotated oppositely and disordered. The photo-induced effect quickly recovered, and some remaining changes recovered for less than 200 ms after turning off the light.

As another example, the predicted results are shown in Fig. S3, S4, and S5 in SI. Figure S3 shows the static condition around topological defects without UV light irradiation. Figure S4 shows the prediction results of the order parameter and the orientation angle, and the summary of these changes is shown in Figure S5.

Figure S3 in SI shows two defects, which are connected by two red and purple brushes. These defects had + 1 and − 1 charges. Throughout the image, the order parameter and orientation angle were predicted from the color information in a, as shown in b and c. In Fig. S3, the regions of the colored brushes showed a lower order parameter (0–0.15) than those of the boundary regions (0.45–0.55). The molecular orientation angle in the purple brushes was 60–80 degrees different from that in the red brush. A drawing of the predicted molecular alignment is summarized as shown in d. The static condition was similar to the first example.

For the same region as Fig. S3, the photo-induced change of the molecular orientation and the order parameter under the UV light irradiation was studied. Similarly, as the first example, the light was irradiated from 0 to 200 ms. For each frame of the movie, the order parameter and the orientation angle were predicted, and the image sequence for the photo-induced change is shown in Fig. S4 (a) and (b) in SI. Same as the previous result, the ordering increased instantly in the purple-brush region, and gradually the orientation angle rotated, while the ordering decreased and the orientational angle rotated in the opposite direction in the red brush gradually. The ordering recovered quickly by turning off the light in red brushes with a minor recovery about 100 ms after light irradiation in the purple-brush region. The recovery was faster than the first example.

The interpretation of this example is shown in Fig. S5. The frustration induced by the photo-isomerization was induced at one pair of the brushes, causing the ordering and the rotation of one type of domain, while the other domain was rotated and disordered by the effect of the residing domain. The photo-induced effect recovered quickly in one domain with a minor recovery for another 100 ms after turning off the light. The photo-induced change was similar to the first example; however, the recovery time was slightly different.

These two examples were presented to show the generality of these phenomena, and we found many similar examples. This study indicates that the frustration stress applied around topological defects is not homogeneous. It is possibly applied to an elastically weaker domain to be ordered, causing the effect to the surrounding domains step by step. This approach, combined with the microscopic observation and the NN function prediction, is a powerful tool to study the local domain change of anisotropic materials like LCs. Since this approach is based on the color dependence of the molecules with different angles and temperatures and the NN function is trained by the same molecule as the one studied, it can be applied to any type of molecule which has the dependence of the refractive index on the angle and the temperature.

## Conclusion

The photo-induced molecular orientation and ordering change were studied by the time-resolved polarization/phase microscopy, combined with a neural network prediction of the orientation angle and order parameter. The neural network function was trained by utilizing the color information of the well-aligned LCs in an alignment cell with various angles and temperatures. We studied the molecular orientation angle/ordering change around topological defects when the photo-induced molecular disordering introduced by light. Microscopic observation and the prediction of the orientation angle and the order parameter revealed local orientation profiles around the topological defects. By light irradiation, the LC molecules in one type of the domain increased in ordering instantly with a gradual rotation of the molecular orientation, causing the disordering and rotation to the surrounding domains. The molecular alignment mostly returned to the initial condition within a few hundreds of milliseconds. The combination of the microscopic observation and the prediction by the NN function is a promising method to reveal the local domain conditions of topological defects. The molecular conditions around the topological defects are inhomogeneous in nature, and various phenomena around them must be clarified locally, and this approach is a powerful tool to study them.

## Supplementary Information

Supplementary Information.
