# Supplementary material for: Inference of molecular orientation/ordering change nearby topological defects by the neural network function from the microscopic color information
Source: Sci Rep. 2021 Apr 27;11:9108. doi: 10.1038/s41598-021-88535-7 (PMC8079417; doi:10.1038/s41598-021-88535-7)
Supplement: Supplementary file 1 — Supplementary Information. [file 41598_2021_88535_MOESM1_ESM.pdf]

Supporting Information

Inference of molecular orientation/ordering change nearby topological defects by the neural network function from the microscopic color information

Haruka Sakanoue[1] Yuki Hayashi[1], and Kenji Katayama[1*]

1 Department of Applied Chemistry, Chuo University, Tokyo 112-8551, Japan;

*Corresponding authors:

K. Katayama, Phone: +81-3-3817-1913, E-mail: kkata@kc.chuo-u.ac.jp

Appendix

Transmittance under the phase/polarization microscope

Under the cross-Nicole condition, no light is transmitted if an LC is in the isotropic state or randomly oriented. Since there is an orientation to an extent around topological defects, patterns of the topological defects can be observed due to the molecular alignment around them.

To understand the color dependence of the transmittance around topological defects, the transmittance spectrum for the cross-Nicole condition is considered. Under the cross-Nicole condition, the transmittance spectrum can be calculated as:

$$\mathrm{I} = \left(\frac{n_3}{n_1}\right) cos^2\alpha \;\; sin^2\alpha\left(t_{\parallel} + t_{\parallel,Shifted} - t_{\perp} - t_{\perp,Shifted}\right)^2 \quad (1)$$

, where n_1 and n_3 are the refractive index of the top and bottom layers for an LC cell, α is the angle of the LC director to the analyzer direction, and $t_{\parallel}$ and $t_{\perp}$ correspond to the Fresnel transmittance coefficients for the light field directions parallel and perpendicular to the molecular axis, respectively. $t_{\parallel,Shifted}$ and $t_{\perp,Shifted}$ are the phase-shifted light by the phase-contrast objective lens for each component. The wavelength dependence comes from the Fresnel transmittance coefficient for a multi-layer system. Under our experimental condition, a thin layer of an LC with a thickness of 3 microns is sandwiched between two glass layers. In this case, the Fresnel transmittance coefficients are given as:

$$t_{\parallel} \text{ or } t_{\perp} = \frac{t_{12}t_{23}\; exp(i\beta)}{1+r_{12}r_{23}\; exp(2i\beta)},\; \beta_e = \frac{2\pi}{\lambda}n_{2,e}(\lambda)d,\; \beta_o = \frac{2\pi}{\lambda}n_{2,o}(\lambda)d \quad (2)$$

, where t_{12}, t_{23}, r_{12} and r_{23} are the Fresnel transmittance and reflectance coefficients at the interfaces between 1 and 2, and 2 and 3, respectively, and λ is the wavelength, $n_{2,e}(\lambda)$, $n_{2,o}(\lambda)$ are the wavelength-dependent extraordinary and ordinary refractive indexes for the LC, respectively, and d is the thickness of the LC layer. This transmittance coefficient, $t_{\parallel}$, $t_{\perp}$ are obtained for the light field parallel and perpendicular to the LC director.

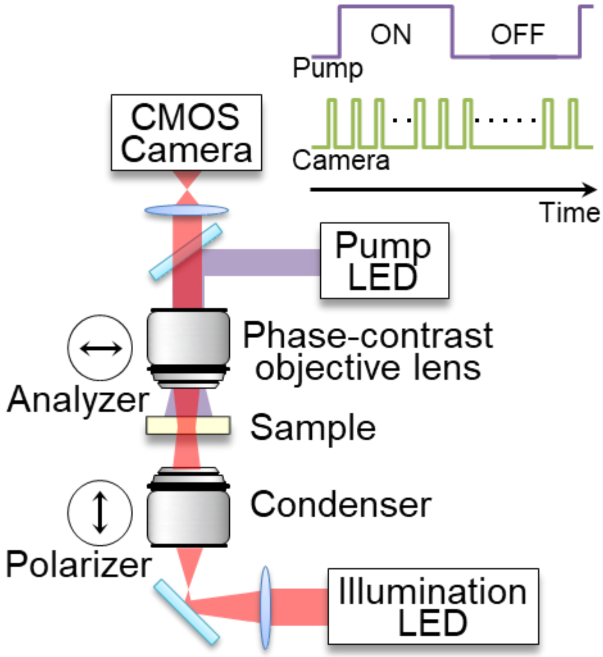

FIGURE. S1 This is the optical configuration of an LED-induced time-resolved phase microscope. The device consists of a phase microscope (BX50, Olympus) with a polarization-dependent detection. A sample was imaged by illuminating a white LED (Thorlabs, SOLIS-3C) from the bottom side under the crossed-Nicole condition. Another pump light with a wavelength of 365 nm (Thorlabs, SOLIS-365C) was illuminated from the top side to the sample after reflected by a dichroic mirror. A sample including photo-responsive molecules absorbed the pump light, causing the molecular orientation change due to the photo-isomerization reaction. The pump light was illuminated to a sample for 200 ms and turned off. The photo-induced molecular orientation change and its recovery were observed during and after the light illumination. Acquisition of an image sequence was started 30 or 20 ms before the illumination of the pump light, and the sequence of images was acquired at a constant interval of 10 ms. Typically, 100 images for 1000 ms were stored for each cycle. After the LC recovered to the original condition, this process was repeated, and the image sequence was stored and averaged several times.

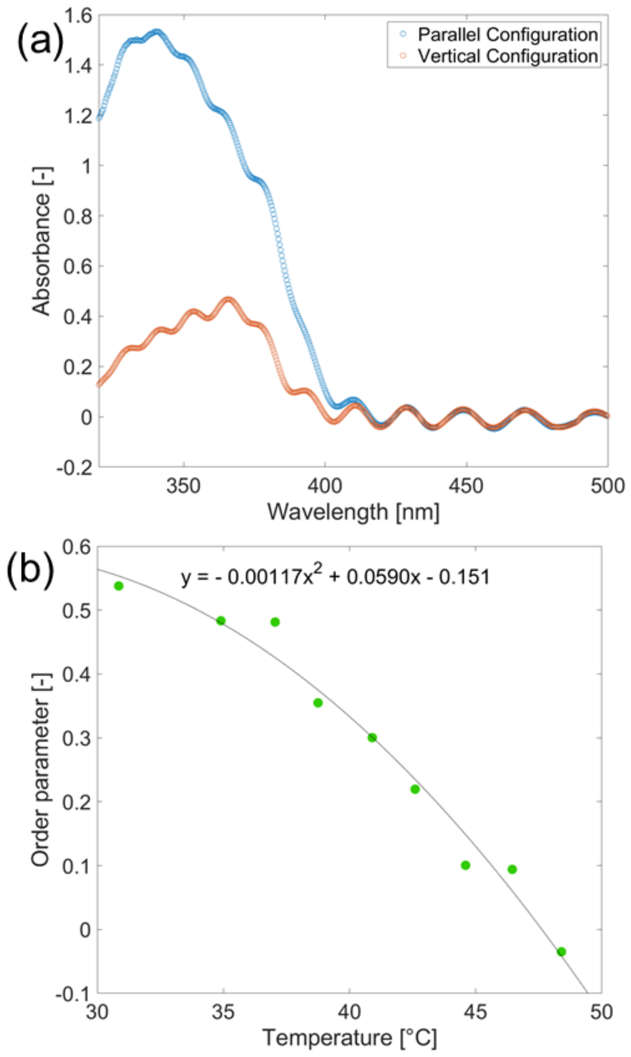

FIGURE. S2 (a) The polarized absorbance spectra for MBBA in a planar alignment cell at 30.9 °C. Blue and red dots show the absorbance when the sample director was parallel and perpendicular to the polarization of the incident light, respectively. (b) The temperature dependence of the order parameter obtained by Equation 1 is shown with a fitting curve. The obtained fitting function was used for conversion from the temperature to the order parameter.

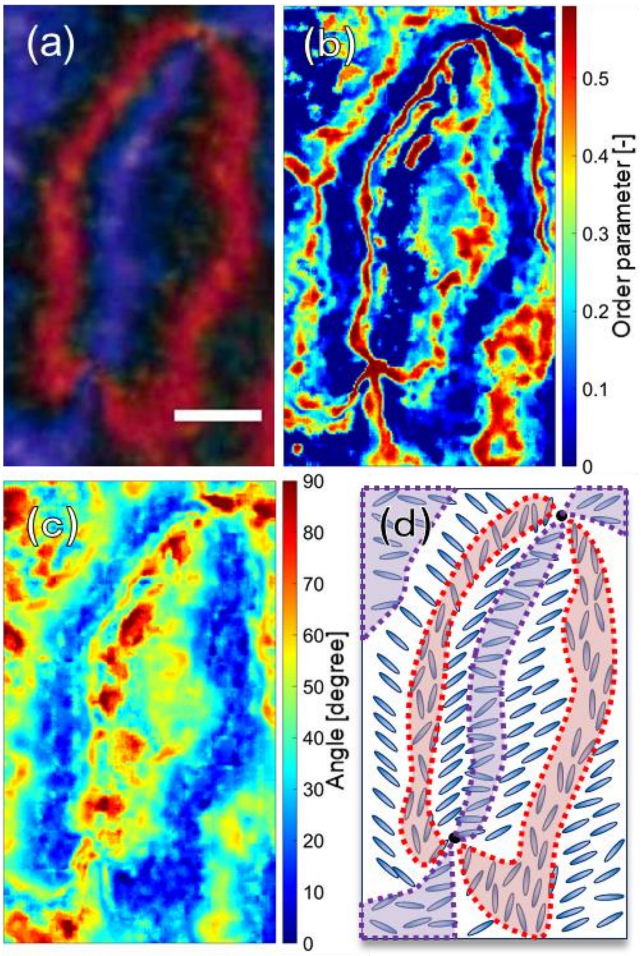

FIGURE. S3 (a) A microscopic image of MBBA before the UV light irradiation is shown. The scale bar corresponds to 20 μm. The predicted image of the order parameter (b) and the orientation angle (c) predicted from the color information of (a) and the neural network function. (d) Based on (b) and (c), the schematic drawing of the molecular ordering and orientation were drawn.

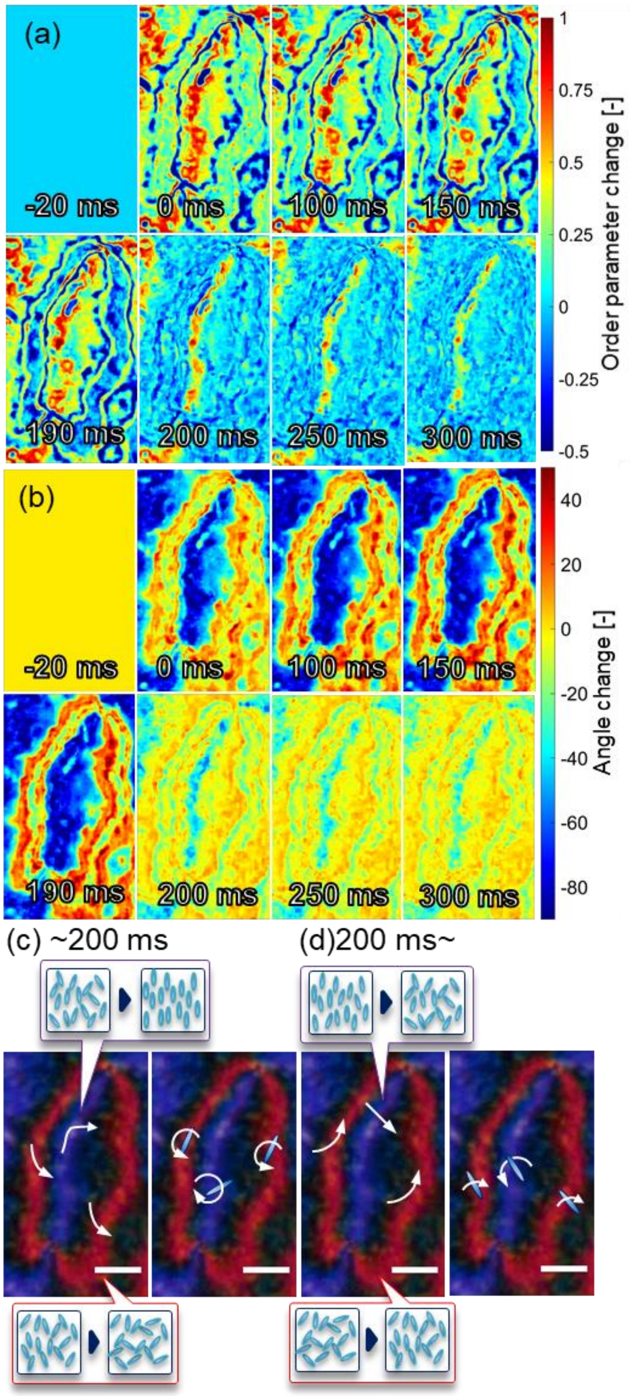

FIGURE.S4 The second example of the prediction of the alignment change under UV light irradiation. The UV light was irradiated from 0 to 200 ms. (a)(b) Time sequences of the prediction of the order parameter and the orientation angle change by irradiation of the UV light. (a) The difference from the original order parameter obtained in Fig. S3(b) and from the initial orientation angle obtained in Fig. S3(c) are shown. (c)(d) The summary figures of the changes in the order parameter (left two images) and the orientation angle (right two images) at each brush region during and after light irradiation.

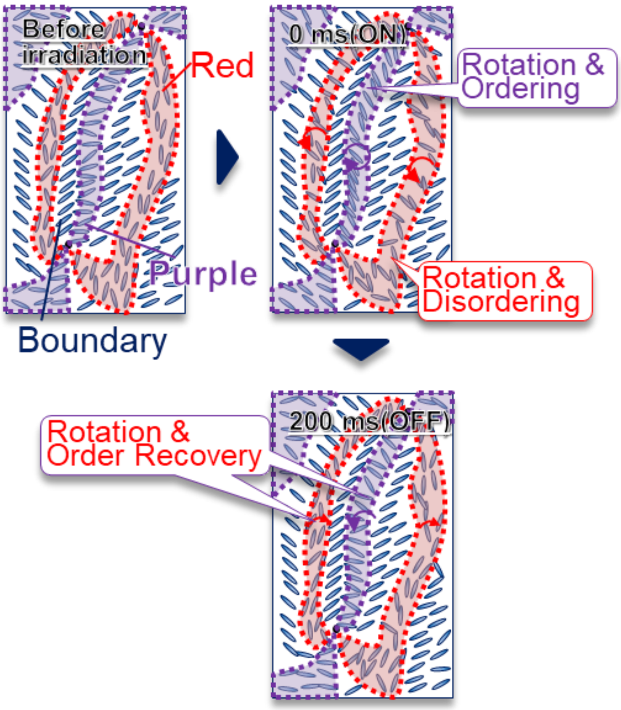

Figure. S5 The schematic drawings of the molecular alignment change before light irradiation (-20 ms), during irradiation (0 ms, 190 ms), and after irradiation (200 ms). The purple and red brush regions corresponded to each colored brush.
